# PD-L1 expression indicates favorable prognosis for advanced lung adenocarcinoma patients treated with pemetrexed

**DOI:** 10.18632/oncotarget.19973

**Published:** 2017-08-07

**Authors:** Pei Zhang, Zhang Bao, Liming Xu, Jianya Zhou, Guohua Lu, Yinan Yao, Rong Liu, Qiqi Gao, Yihong Shen, Jianying Zhou

**Affiliations:** ^1^ Department of Respiratory Diseases, The First Affiliated Hospital, College of Medicine, Zhejiang University, Hangzhou, China; ^2^ Department of Pathology, The First Affiliated Hospital, College of Medicine, Zhejiang University, Hangzhou, China

**Keywords:** programmed death ligand 1, pemetrexed, immunohistochemistry, lung adenocarcinoma

## Abstract

Conventional chemotherapy for lung cancer exerts anti-tumor effects through cytotoxicity, and through immunologic regulation by reducing specific T cell subsets and inducing the expression of programmed death ligand 1 (PD-L1) on tumor cells. Even though pemetrexed has shown huge potential in combination with other targeted or immune therapies, there is still little information about the values of specific immune checkpoint markers for advanced lung adenocarcinoma treated with pemetrexed. In the present study, a total of 56 patients with advanced lung adenocarcinoma, who received pemetrexed-based chemotherapy, were included retrospectively. Immunohistochemistry was performed to assess PD-L1, programmed death 1 (PD-1), thymidylate synthase, and tumor infiltrating lymphocytes (TILs). In this cohort, the positive expression of PD-L1 and PD-1 were 26.8% and 33.9% respectively. PD-L1, PD-1, and thymidylate synthase expression were not significantly associated with any clinical features, while the expression of both PD-L1 and PD-1 were correlated with Ki-67 expression. Furthermore, the expression of PD-1 was significantly correlated with TILs. The progression-free survival (PFS) in patients with PD-L1^+^ specimens was significantly longer compared to PD-L1^−^ specimens. Moreover, PD-L1 expression was an independent protective factor for PFS, and the smoking status was an independent risk factor. PD-L1 expression was significantly associated with better prognosis for patients with pemetrexed-based treatment. Our findings suggested that PD-L1 expression might be a favorable prognostic biomarker for pemetrexed-based regimen, which is a rationale for combining immunotherapy with chemotherapy for lung cancer.

## INTRODUCTION

Lung cancer is the leading cause of cancer-related mortality worldwide, causing more than one million deaths annually [1]. Approximately 85% of patients with lung cancer are diagnosed with non-small-cell lung cancer (NSCLC), while 80% are diagnosed with advanced stages [2, 3]. For decades, conventional cytotoxic chemotherapy, which includes platinum (cisplatin or carboplatin) and a non-platinum drug (pemetrexed, gemcitabine, etc.), has been used as the standard treatment in these patients [4]. Even though, the great progress has recently been made in targeted therapies and immunotherapies, platinum-based doublet chemotherapy still remains the foundation for the majority of patients with advanced lung cancer [4]. Furthermore, chemotherapy has been shown to regulate the immune system and overcome the resistance to targeted therapy, indicting its potential for coordination with other therapies [4]. However, the predictive biomarkers for chemotherapy and its combination strategy remain unknown.

Pemetrexed, a potent inhibitor of thymidylate synthase (TS) and other folate-dependent enzymes involved in purine and pyrimidine synthesis, has been approved as the first-line, second-line, and maintenance treatment for patients with non-squamous NSCLC [5, 6]. Moreover, due to its better tolerability and lower toxicity compared with other cytotoxic agents, pemetrexed shows huge potential in combination with other targeted or immune therapies [6]. It has been reported that in patients treated with pemetrexed, increased expression of TS was associated with resistance to TS-targeting drugs, while low expression of TS was related to better clinical outcomes [7]. However, other studies suggested that the association between TS expression and efficacy of pemetrexed was not relevant [8]. Interestingly, pemetrexed was also reported to cause inflammation of seborrheic keratosis and scleroderma-like conditions in patients with lung cancer, which further revealed that interleulin-4 and interleulin-6-associated-T cells were involved in this pathogenesis [9, 10]. Thus, it is essential to explore potential immune-related biomarkers for patients treated with pemetrexed.

The initiation and progression of lung cancer is a dynamic process, monitored by the immune system, where both myeloid and lymphoid populations control the immune responses to neoplasm [11]. In tumor microenvironment, activity of infiltrated T cells is inhibited by programmed death protein 1/programmed death ligand 1 (PD-1/PD-L1) pathway (referred to as the PD pathway), which acts as a specific immune checkpoint pathway [12]. Immune checkpoints refer to multiple inhibitory pathways that counteract certain crucial steps of T cell-mediated immunity to maintain self-tolerance and modify the duration and amplitude of immune responses [13-15]. PD-1, a 288 amino acid cell surface protein molecule, has two ligands, PD-L1 (also known as B7-H1 and CD274) and PD-L2 (also known as B7-DC and CD273). In the past few years, PD-1-targeting monoclonal antibodies such as nivolumab and pembrolizumab, have shown great promises and to be well tolerated [12]. Furthermore, several clinical trials using nivolumab or pembrolizumab combined with standard chemotherapy have showed encouraging results in patients suffering with different types of cancer [16, 17]. Antibodies targeting PD-L1, such as atezolizumab, durvalumab, and avelumab, have been developed and evaluated, revealing promising results in terms of efficacy and safety [18, 19]. The association between abnormal PD-L1 expression and NSCLC survival has been investigated, nonetheless the results remain controversial [20-22]. In addition, it has been proposed that tumor microenvironment could be stratified into four groups based on conditions of tumor infiltrating lymphocytes (TILs) and PD-L1 expression [15]. Interestingly, a recent randomized, multicohort study (KEYNOTE-021) demonstrated that combination of pembrolizumab, carboplatin, and pemetrexed could be an effective and tolerable first-line treatment option for patients with advanced NSCLC [23], suggesting there might be correlation of PD-L1 expression and pemetrexed-based chemotherapy. Therefore, it may be interesting to investigate the possible predictive values of the PD pathway and TILs in lung adenocarcinoma patients treated with pemetrexed.

The purpose of the current study is to investigate the possible association between the PD pathway and the prognosis for lung adenocarcinoma patients treated with pemetrexed-based chemotherapy. We found that PD-L1 expression was significantly correlated with a longer survival in these patients, indicating that the expression of PD-L1 might be a favorable prognosis biomarker for pemetrexed-based therapy, which is a rationale for combining the immunotherapy with chemotherapy for lung cancer.

## RESULTS

### Patients characteristics

From January, 2009, to December, 2015, a total of 56 patients (37 male and 19 female) were enrolled in this cohort study. Clinical characteristics of registered patients are shown in Table 1. Most of these patients were diagnosed with staged IV (85.7%) lung cancer. For these 56 patients, 17 patients showed partial response, 34 patients showed stable disease, while only 5 patients showed disease progression, after two cycles of pemetrexed-based chemotherapy. The objective response rate (ORR) and disease control rate (DCR) to chemotherapy were 30.4% and 91.1%, respectively (Table 2).

**Table 1:** Baseline demographics and disease characteristics

Characteristics	No. of patients (%)
Gender	
Male	37 (66.1)
Female	19 (33.9)
Age (years)	
Median	58
Range	29-79
<58 years	22 (39.3)
≥58 years	34 (60.7)
Smoking status	
Former or current smoker	33 (58.9)
Never smoker	23 (41.1)
ECOG performance status	
0	15 (26.8)
1	27 (48.2)
2	14 (25.0)
Stage	
Stage IIIB	8 (14.3)
Stage IV	48 (85.7)
Ki-67 positivity	
≤15%	24 (42.9)
>15%	32 (57.1)

ECOG = Eastern Cooperative Oncology Group.

**Table 2:** Responses assessed by RECIST version 1.1

Patients (n)	CR (n)	PR (n)	SD (n)	PD (n)	ORR (%)	DCR (%)
56	0	17	34	5	30.4	91.1

RECIST = Response Evaluation Criteria in Solid Tumors; CR = complete response; PR = partial response; SD = stable disease; PD = disease progression; ORR = overall response rate; DCR = disease control rate.

### The expression of PD-L1, PD-1 and TS examined by immunohistochemistry

Immunohistochemistry (IHC) assay was used to determine the incidence and patterns of PD-L1, PD-1 and TS in the tumor tissue. In our cohort of 56 patients, the percentage of patients with PD-L1^+^ and PD-1^+^ tumors was 26.8% (15 of 56) and 33.9% (19 of 56), respectively. In addition, there were only three patients who expressed both PD-L1 and PD-1. Meanwhile, the percentage of TS^+^ was 46.4% (26 of 56), which was much higher compared to PD-L1 and PD-1 expression (Table 3).

**Table 3:** PD-L1, PD-1 and TS expression in pemetrexed-treated patients

Patients (n)	PD-L1	PD-1	TS
Positive (n)	15	19	26
Negative (n)	41	37	30

PD-L1 = programmed death ligand 1; PD-1 = programmed death protein 1; TS = thymidylate synthase.

**Figure 1:**
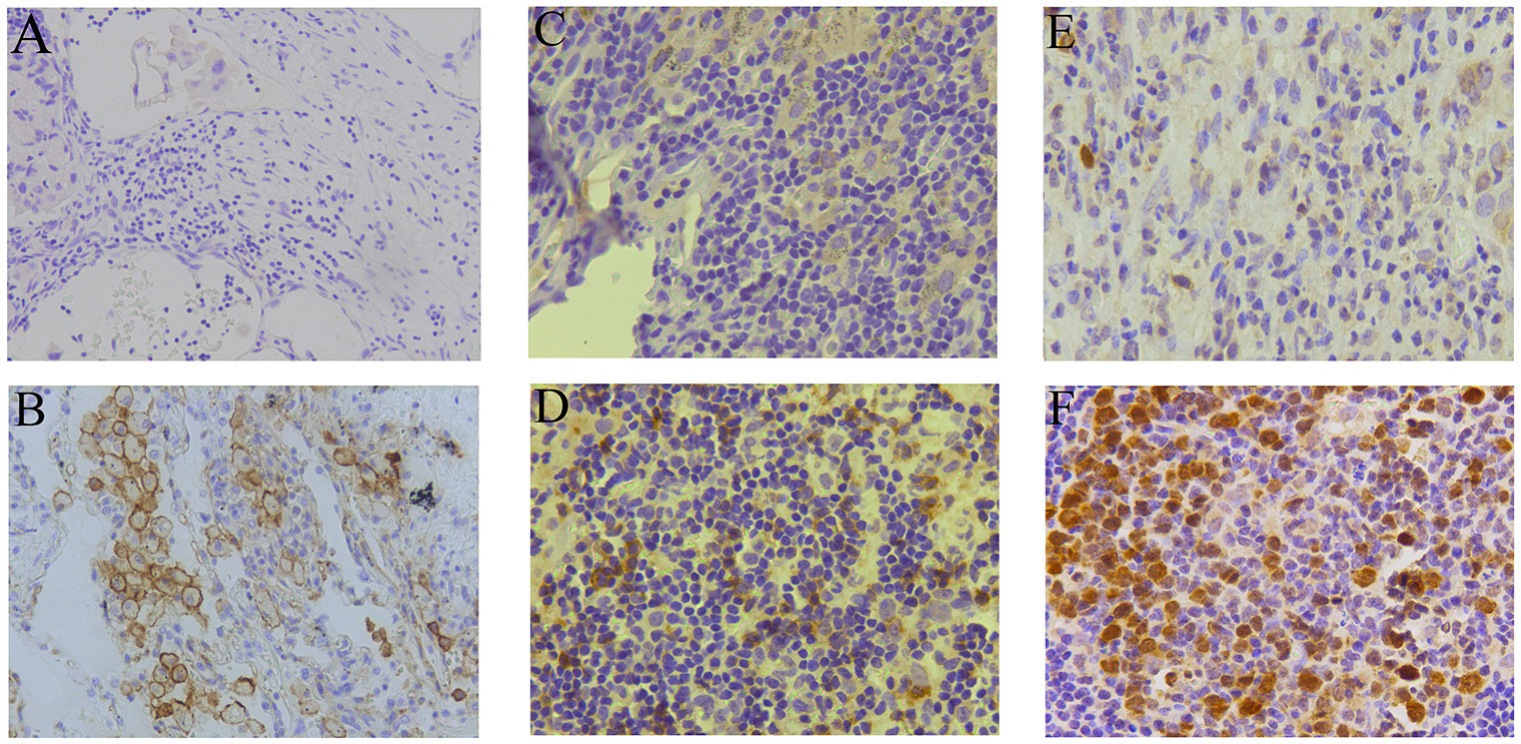
PD-L1, PD-1, and TS immunohistochemistry analysis Representative immunohistochemistry images of PD-L1 negative **(A)** and positive **(B)** expression, PD-1 negative **(C)** and positive **(D)** expression, TS low **(E)** and high **(F)** expression in tumor specimens. Magnification, 200×.

Additionally, we found that PD-L1 was expressed primarily on the cell membrane. Moreover, PD-L1 was expressed not only on tumor cells, but also on immune-infiltrating cells, including TILs, associated histocytes, and macrophages. Compared to PD-1^−^ samples, PD-1 expression was mostly found in lymphocytes within the stroma of PD-1^+^ tumors. In addition, the staining for TS appeared in both nuclei and cell cytoplasm (Figure 1).

### Relation between PD-L1, PD-1, TS expression and clinical characteristics

To investigate the relationship between the expression of PD-L1, PD-1, TS and the clinicopathologic features of patients with advanced lung adenocarcinoma, who received pemetrexed-based treatment, the χ^2^ test was performed. Neither the expression of PD-L1 nor PD-1 was correlated with any clinical characteristics, including gender, age, smoking status, performance status, and tumor stage (Table 4). PD-L1 positivity was significantly related to high expression of Ki-67 (>15%), indicating high cell proliferation and tumor progression in this population. Furthermore, PD-1 positivity was also correlated with high Ki-67 expression (Table 4), suggesting orchestrating effects in the PD pathway. In addition, the expression of TS was not associated with any of clinical features or the Ki-67 expression (Table 4).

**Table 4:** Relation between expression of PD-L1, PD-1, TS and clinical characteristics

Characteristic	Patients (n = 56)	PD-L1			PD-1			TS		
		Positive (n = 15)	Negative (n = 41)	*P* value^a^	Positive (n = 19)	Negative (n = 37)	*P* value^a^	Positive (n = 26)	Negative (n = 30)	*P* value^a^
Gender				0.064			0.354			0.642
Male	37	7	30		11	26		18	19	
Female	19	8	11		8	11		8	11	
Age (years)				0.581			0.143			0.906
<58 years	22	5	17		10	12		10	12	
≥58 years	34	10	24		9	25		16	18	
Smoking status				0.259			0.067			0.861
Former or current smoker	33	7	26		8	25		15	18	
Never smoker	23	8	15		11	12		11	12	
Performance status				0.223			0.625			0.757
0-1	42	13	29		15	27		19	23	
2	14	2	12		4	10		7	7	
Stage				0.460			0.565			0.827
Stage IIIB	8	3	5		2	6		4	4	
Stage IV	48	12	36		17	31		22	26	
Ki-67 positivity				0.025			0.011			0.832
≤15%	25	3	22		4	21		12	13	
>15%	31	12	19		15	16		14	17	

PD-L1 = programmed death ligand 1; PD-1 = programmed death protein 1; TS = thymidylate synthase.

a: For this analysis, χ^2^ test and Yates’ correction were applied.

### Relation between PD-L1, PD-1 expression, and TILs in advanced lung adenocarcinoma

Although the immune responses within the tumor microenvironment are widely implicated as a determining factor in tumor progression and aggressiveness, the recent positive results with immune checkpoint inhibitors in lung cancer have created new interest in TILs and their relationship to tumor immunity and chemotherapy response [24, 25]. In this study, we evaluated the intratumoral relation between PD-L1, PD-1 expression and TILs by IHC staining for CD4 and CD8. Although some specimens had CD4^+^ and CD8^+^ TILs in both PD-L1^+^ and PD-L1^−^ tumors, no significant difference between PD-L1 expression and the scores of CD4^+^ or CD8^+^ TILs was found in our study (Table 5). However, we found that the PD-1 expression was related to both CD4^+^ and CD8^+^ TILs in the patients with advanced lung adenocarcinoma (Table 5).

**Table 5:** Relation between PD-L1, PD-1 and TILs scores in tumors

Patients (n)	CD4^+^ TILs					CD8^+^ TILs				
	Score (n)					Score (n)				
	0	1	2	3	*P* value^a^	0	1	2	3	*P* value^a^
PD-L1					0.113					0.430
Positive (n = 15)	2	5	5	3		12	3	0	0	
Negative (n = 41)	11	18	7	5		29	9	3	0	
PD-1										
Positive (n = 19)	2	7	5	5	0.026	9	7	3	0	0.001
Negative (n = 37)	11	16	7	3		32	5	0	0	

PD-L1 = programmed death ligand 1; PD-1 = programmed death protein 1; TILs = tumor-infiltrating lymphocytes.

a: For this analysis, Kruskal-Wallis test was applied.

### Relation between PD-L1, PD-1, TS expression and clinical responses of patients with advanced lung adenocarcinoma

We further investigated the relationship between the PD pathway and the therapeutic responses of patients treated with pemetrexed-based chemotherapy by the Kaplan-Meier method. Among a total of 56 patients, the median progression-free survival time was 4.4 months (range from 1.6 to 22.2 months). In our cohort of 56 patients, who received pemetrexed-based treatment as first-line chemotherapy, the ORR and DCR of pemetrexed have not shown significant difference with PD-L1 and PD-1 expression (Table 6). Moreover, no significant difference was observed between the ORR and DCR of pemetrexed and the TS expression (Table 6). In addition, among 56 patients, only 5 patients evaluated disease progression after two cycles’ chemotherapy, and they were all PD-L1^−^.

**Table 6:** Relation between PD-L1, PD-1, TS expression and clinical outcomes

Patients (n)	ORR (n = 17)		DCR (n = 51)	
	n (%)	*P* value^a^	n (%)	*P* value^a^
PD-L1		0.716		0.378
Positive (n = 15)	4 (26.7)		15 (100.0)	
Negative (n = 41)	13 (31.7)		36 (87.8)	
PD-1		0.278		0.846
Positive (n = 19)	4 (21.1)		18 (94.7)	
Negative (n = 37)	13 (35.1)		33 (89.2)	
TS		0.950		0.268
Positive (n = 26)	8 (30.8)		22 (84.6)	
Negative (n = 30)	9 (30.0)		29 (96.7)	

PD-L1 = programmed death ligand 1; PD-1 = programmed death protein 1; TS = thymidylate synthase; ORR = overall response rate; DCR = disease control rate.

a: For this analysis, χ^2^ test and Yates’ correction were applied.

**Figure 2:**
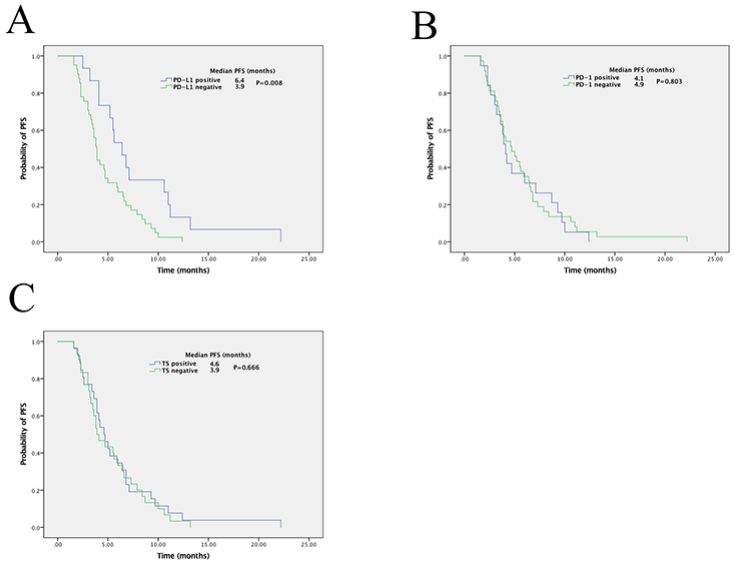
Kaplan-Meier curves of progression-free survival (PFS) in patients with pemetrexed-treated lung adenocarcinoma PD-L1 expression **(A)**, PD-1 expression **(B)**, and TS expression **(C)** of patients in correlation with PFS. The *P* value for the difference between the two was determined by the log-rank test.

The Kaplan-Meier method was used to evaluate the survival of patients (Figure 2). In the cohort of 56 patients, the PFS of patients with PD-L1^+^ specimens was significantly longer than that of PD-L1^−^ specimens (median FPS, 6.4 *vs.* 3.9 months; *P* = 0.008; Figure 2A). In contrast, PD-1 and TS expression did not show significant correlation with PFS (PD-1, 4.1 *vs.* 4.7, *P* = 0.803; TS, 4.6 *vs.* 3.9, *P* = 0.666; Figure 2B and 2C). To conclude, these results indicated that PD-L1 might be a potential predicative factor for treatment efficacy of pemetrexed in patients with advanced adenocarcinoma.

### Relation between clinical factors, immunohistochemical findings, and patient survival times

Multivariate Cox regression was used to analyze protective factors for pemetrexed-based chemotherapy in lung cancer patients. Never smoker patients had much longer PFS compared to former or current smokers (HR = 7.937, *P* = 0.011, Table 7), suggesting that smoking status was an independent risk factor for PFS. Consistent with previous results, PD-L1 expression was also revealed as an independent protective factor for PFS (HR = 0.193; *P* = 0.001, Table 7), implying that patients with PD-L1 positive staining might have a better response to pemetrexed-based chemotherapy.

**Table 7:** Multivariate analyses of prognosis factors in association with patient survival times

Characteristic	PFS	
	HR (95% CI)	*P* value
Gender (male *vs.* female)	0.579 (0.487-6.113)	0.398
Age (<58 *vs.* ≥58 years)	1.108 (0.555-2.212)	0.772
Smoking (yes *vs.* no)	7.937 (0.026-0.621)	0.011
PS		0.380
PS (1)	1.982 (0.756-5.200)	0.164
PS (2)	1.450 (0.632-3.324)	0.380
Stage (IV *vs.* IIIB)	0.883 (0.343-2.275)	0.797
Ki-67 (≤15% *vs.* >15%)	0.931 (0.436-1.988)	0.854
PD-L1 (positive *vs.* negative)	0.193 (2.018-13.342)	0.001
PD-1 (positive *vs.* negative)	1.460 (0.298-1.575)	0.373
TS (positive *vs.* negative)	0.873 (0.576-2.278)	0.698

HR = hazard ratio; CI = confidence interval; PFS = progression-free survival; PS = performance status; PD-L1 = programmed death ligand 1; PD-1 = programmed death protein 1; TS = thymidylate synthase.

## DISCUSSION

This study retrospectively reviewed a cohort of advanced lung adenocarcinoma patients, who were treated with first-line pemetrexed-based chemotherapy. PD-L1/PD-1 expression in the specimen was assessed, and correlations between the efficacy of treatment and the PD pathway were analyzed. For these 56 patients, the ORR and DCR to pemetrexed-based therapy were 30.4% and 91.1% respectively, which is consistent with previous clinical trials [26, 27]. These results confirmed pemetrexed as highly effective agent in advanced lung adenocarcinoma. The expression of PD-L1 was 26.8% (15 of 56) in all the tumor specimens, while percentage of PD-1 expression was 33.9% (19 of 56). The frequency of PD-L1 expression in primary NSCLC specimens showed conflicting data. The incidence of PD-L1 expression in the overall population of patients with NSCLC has shown to be 30% to 50% [22, 28, 29]. A variety of PD-L1 immunohistochemistry antibodies, including SP142, SP263, 22C3, E1L3N, and 28-8 clones, have been utilized in clinical trials of immune checkpoint inhibitors [28, 29]. Furthermore, there has been no consensus on the PD-L1 standard antibody and threshold definition for positive staining [28, 29]. Uses of PD-L1 antibodies, varying in their specificity and sensitivity, as well as the cut-off definition could explain these conflicting results in literature [30, 31]. Different thresholds (e.g. 5%, 25%, and 50%) of PD-L1 expression in tumors cells have shown to be associated with an increased likelihood of response to PD-1 targeting checkpoint inhibitors [24, 32]. We chose a PD-L1 antibody with SP142 clone, which has much lower positivity rate compared with 22C3 and 28-8 clones. Consequently, cut-off of 5% membranous immunohistochemical signal on tumor cells was empirically chosen as the threshold in our study.

We further examined relationship between TILs infiltration and expression of PD-L1 and PD-1. Our results suggested there was no correlation between PD-L1 expression and the scores of CD4^+^ or CD8^+^ TILs, nevertheless the expression of PD-1 was significantly correlated with both CD4^+^ and CD8^+^ TILs in patients with advanced lung adenocarcinoma. Previously, researches have demonstrated that increased PD-1 expression on peripheral blood CD8^+^ T cells was associated with impaired immune function in patients with NSCLC, and with an increasing intensity of immune infiltrates and the presence of lymphoid aggregates [32, 33]. Contrary, PD-L1 expression appears to have a negative effect on the host’s antitumor response in metastatic melanoma, and there have been little evidence to show the correlation between PD-L1 expression and TILs in NSCLC [34]. Based on the obtained data, we inferred that TILs were much more important for driving PD-1 expression compared to PD-L1 in advanced lung adenocarcinoma, nonetheless their presence alone was not sufficient to reverse compromised immune surveillance in tumor.

Although the efficacy of targeted therapies has been proven in numerous trials, platinum-based doublet chemotherapy remains therapeutic foundation for advanced NSCLC patients without actionable genetic alterations. Predictive biomarkers are crucial for decisions regarding the use of specific molecular or cytotoxic agents. Pemetrexed has been proven to be the most effective cytotoxic agent in lung adenocarcinoma patients. The principal target of pemetrexed, a multi-targeted antifolate that gains entry to the cell via reduced folate carrier, is TS. TS is responsible for the conversion of deoxyuridine monophosphate to deoxythymidine monophosphate necessary for DNA replication. Existing studies have suggested a potential association between overexpression of TS and reduced sensitivity to pemetrexed in antifolate-resistant cell lines [35]. Resistance to pemetrexed was significantly associated with increasing TS gene expression [36], while TS appeared as a strong predictive marker for pemetrexed sensitivity. Different studies argued that the association between TS expression and clinical results was not statistically significant [8]. Our results showed no significant difference between TS expression and clinical responses, potentially due to the limited patient number in the cohort. In addition, the optimal and standard assay methods for TS expression still need to be established.

In recent years, various biological markers, such as the nucleotide excision repair pathway, cell-cycle regulators, β-tubulin class III, epidermal growth factor receptor mutations, and gene expression profiling, have been explored to predict the efficacy of traditional chemotherapies in patients with lung cancer [37, 38]. To further explore the relationship between the PD pathway and the therapeutic response or survival of pemetrexed-treated patients, we performed the Kaplan-Meier method and multivariate Cox regression analysis. Our results showed that in the cohort of 56 patients, the PFS of patients with PD-L1^+^ specimens were significantly longer compared to PD-L1^−^ specimens. Meanwhile, the Cox regression analysis has shown that PD-L1 expression was an independent protective factor for PFS, suggesting patients with PD-L1 positive staining might have a better response to pemetrexed-based treatment. However, the expression of PD-1 did not show any significant correlation with PFS. The PD-L1 expression has emerged as a seemingly promising biomarker for NSCLC patients, but the relationship between PD-L1 expression and prognosis in patients with NSCLC remains controversial. A study based on a large sample size demonstrated that patients with NSCLC and PD-L1 expression (both mRNA and protein) beyond the detection threshold had significantly better outcomes in 2 separate cohorts [31]. Previous studies have linked negative PD-L1 expression with superior overall survival (OS) in NSCLC patients compared to patients with positive PD-L1 expression [20, 39, 40], while others concluded that PD-L1 expression had no significant correlation with OS but is related to longer relapse-free survival [41]. It is noteworthy that most previous researches have been performed in the overall population of patients with NSCLC, which is differed from our present study. Our study was designed to specifically target a cohort of patients with advanced lung adenocarcinoma who received pemetrexed-based chemotherapy. This was also the first study to explore the relationship between clinical outcomes of pemetrexed-based treatment and PD-1, PD-L1 expression. In addition, we found that the expression of both PD-L1 and PD-1 were significantly associated with high Ki-67 expression, indicating the potential connection between the PD-1 pathway and tumor proliferation. Therefore, we draw a conclusion that PD-L1 expression might represent a favorable prognostic marker for patients with advanced lung adenocarcinoma, who receive pemetrexed-based chemotherapy.

In the multivariate analyses of prognostic factors for patient survival, we found the smoking status to be an independent risk factor for PFS. Moreover, PD-L1 expression was an independent protective factor for PFS, which indicated that the expression of PD-L1 is a predictive biomarker for response to pemetrexed-based treatment. These results cast light on potential suitable markers for patients with lung cancer who might benefit from pemetrexed therapy.

Cancer immunotherapy, which differs from traditional cytotoxic therapies and targeted therapies, is a new class of anti-cancer drugs with promising clinical activity in different types of tumors [18, 42-45]. Previous studies have reported that pemetrexed-based chemotherapy in anaplastic lymphoma kinase rearrangement patients was associated with a higher response rate and longer PFS [46]. The combination of pembrolizumab, carboplatin, and pemetrexed provides a significant and clinically relevant improvement in antitumor activity compared with chemotherapy alone [23]. Different clinical trials suggested that nivolumab plus platinum-based doublet chemotherapy may improve outcomes and extend patients survival in advanced NSCLC in the first-line setting [47]. We demonstrated that the expression of PD-L1 was associated with pemetrexed-based chemotherapy, which might help to guide patient selection and therapeutic optimization.

The limitations in our study include the limited sample size of the study cohort, which might have reduced the statistical power of the analysis, although we have verified and improved the testing methods. What’s more, the study was a retrospective single-center study, meaning it might lead to potential follow-up bias and selection bias. To consolidate our findings, further studies should be multicenter with large sample sizes.

In summary, the present study is the first to demonstrate the relationship between PD-L1 expression and the survival for patients with advanced lung adenocarcinoma who received pemetrexed-based therapy. Our results indicated that PD-L1 expression was significantly correlated with a longer PFS in patients with advanced lung adenocarcinoma following pemetrexed-based treatment, and PD-L1 expression was an independent protective factor for PFS. Furthermore, the expression of both PD-L1 and PD-1 were associated with Ki-67 expression in significant correlates. To conclude, our study suggests that PD-L1 expression might be a favorable prognosis biomarker for pemetrexed. It also provides a rationale for combining the immunotherapy with chemotherapy in the future.

## MATERIALS AND METHODS

### Study population

A total of 145 patients diagnosed with advanced lung adenocarcinoma (stage IIIB-IV) from The First Affiliated Hospital of Zhejiang University, from January, 2009 to December, 2015 (Union for International Cancer Control, TNM classification of malignant tumors, 7^th^ edition) [48], were included in this study. All patients received pemetrexed-based (Alimta^®^, Lilly France S.A.S) chemotherapy as the first-line treatment. Patients who had not been initially diagnosed with an advanced stage (stage III-IV), or who had received chemotherapy with a concurrent surgical procedure or radiotherapy, were excluded from the study. Of these 145 patients, 56 primary biopsy tumor samples were obtained for further tissue analysis. Clinical and pathologic characteristics were collected using retrospective chart review. This study was approved by the Institutional Review Board of the First Affiliated Hospital of Zhejiang University.

### Patients treatment and assessments of patient clinical features

A baseline assessment of clinical features including gender, age, smoking status, performance status, and tumor stage were retrospectively reviewed using medical records. All patients received intravenous injection of pemetrexed in combination with cisplatin, or carboplatin, or nonplatinum agents, at a dose of 500 mg/m^2^ body surface area, every 21 days; additionally, all patients received folic acid, vitamin B_12_, and dexamethasone in order to avoid adverse drug reactions. Therapy was continued until clinical or radiological disease progression, or until intolerable toxicity was observed, or in case of patients’ death. The ORR and DCR were evaluated every two cycles, or earlier, or in case clinical signs of progression were observed, according to the Response Evaluation Criteria in Solid Tumors version 1.1 [49]. PFS was defined as the time from pemetrexed treatment commencement to the first documented radiologic/clinical disease progression or death from any cause.

### Immunohistochemistry

The biopsy and surgical resection specimens were fixed in formalin and embedded in paraffin using standardized procedures. Deparaffinized sections (4 μm) were stained using standardized IHC procedures [50]. Briefly, endogenous peroxidase activity was blocked by using hydrogen peroxidase for 10 minutes; heat retrieval with sodium citrate buffer (for demasking antigen), and ethylenediaminetetraacetic acid repair were performed before IHC. After washing with phosphatebuffered saline, tissue sections were incubated with primary antibodies at room temperature for 15 and 60 minutes, respectively. After additional washing, the reaction was visualized with 3,3’ diaminobenzidine tetrahydrochloride. A rabbit anti-PD-L1 monoclonal antibody (clone SP142, ZSGB-BIO, Beijing, China), a mouse anti-PD-1 monoclonal antibody (clone UMAB199, ZSGB-BIO, Beijing, China), and a mouse anti-TS monoclonal antibody (clone 4H4B1, Invitrogen, Carlsbad, CA) were used. The sections were counterstained with hematoxylin. Isotype-matched IgG was used as a control. The expression of CD4 (clone UMAB64, ZSGB-BIO, Beijing, China) and CD8 (clone SP16, ZSGB-BIO, Beijing, China) were also performed to assess CD4^+^ and CD8^+^ TILs according to the standard automated protocols. In addition, we also evaluated Ki-67, which is a nuclear protein associated with cellular proliferation, as previously described [51].

### Scoring

All samples were independently scored by two experienced pathologists (Liming Xu and Qiqi Gao), who were blinded to patients’ clinical characteristics and outcomes. The expression of PD-1 was quantified using a visual grading system, with the staining intensity graded as 0 to 3 (0, absent; 1, moderate; 2, intermediate; 3, strong). The number of positive cells was graded using a 0 to 3 scale (0, 0%; 1, 1%-10%; 2, 11%-50%; 3, 51%-100%) [50]. The scores for both intensity and proportion were multiplied to obtain the final semiquantitative H-score. The mean H-score from all the patient samples was used as the cut-off value to determine the PD-1 positivity. PD-L1 expression by tumor cells and TILs was scored at 5% interval. Specimens with ≥ 5% membranous expression were considered positive [52]. The tumor sections stained for CD4 and CD8 were evaluated for the intensity of TILs, which was judged to be 0 to 4 (0, absent; 1, sparse; 2, scattered; 3, dense; 4, very dense) [53]. The positivity of either CD4^+^ or CD8^+^ TILs was defined as score of ≥ 1. The expression of TS was quantified using the H scoring method defined as described in literature [7]. The mean H-score from all the patient samples was used as the cut-off value to determine TS positivity. In addition, a cut-off value of 15% was defined as Ki-67 positivity [51].

### Statistical analysis

Correlations between PD-1, PD-L1 and TS expression and various clinicopathologic characteristics were analyzed using the χ^2^ test and Yates’ correction. We examined the correlation between proportion of tumor cells expressing PD-L1 or PD-1 and the intensity of immune cell infiltration using the Kruskal-Wallis rank test. The Kaplan-Meier method was used to estimate the survival probability, while the log-rank test to determine the statistical significance. Multivariate analysis using the Cox regression model was conducted to analyze the clinicopathologic features. All tests were 2-sided. *P* < 0.05 was considered statistically significant. The statistical analyses were performed using the SPSS software package, version 19.0 (IBM, Armonk, NY).
